# Investigation of Histamine Removal by Electrodialysis from the Fermented Fish Sauce and Its Effects on the Flavor

**DOI:** 10.3390/foods12061325

**Published:** 2023-03-20

**Authors:** Yicheng Ding, Mengting Qiu, Xiaoling Tang, Renchao Zheng, Xuxia Zhou

**Affiliations:** 1 Key Laboratory of Marine Fishery Resources Exploitment & Utilization of Zhejiang Province, College of Food Science and Technology, Zhejiang University of Technology, Hangzhou 310014, China; 2 Key Laboratory of Bioorganic Synthesis of Zhejiang Province, College of Biotechnology and Bioengineering, Zhejiang University of Technology, Hangzhou 310014, China

**Keywords:** fish sauce, electrodialysis, response surface methodology

## Abstract

Histamine is one of the most concerned safety indicators in fish sauce. Considering its charge property, electrodialysis (ED) was used to control the histamine in fish sauce, and studies were focused on three operating parameters: input current, pH, and flow velocity. A Box–Behnken design and response surface methodology was adopted to derive a statistical model, which indicated that 5.1 A input current, pH 3.8, and 40 L∙h^−1^ flow velocity were optimal operation conditions. Under this condition, the histamine removal rate reached 53.41% and the histamine content met the allowable histamine limit of below 400 mg·kg^−1^ in fish sauce, while the amino nitrogen (ANN) loss rate was only 15.46%. In addition, amino acids and volatile compounds changed differently during ED. As a result, with decreased histamine, the fish sauce after ED was also less salty and less fishy. The study first explored utilizing ED to remove histamine from fish sauce, which has positive implications for promoting the safety of aquatic products.

## 1. Introduction

Fish sauce is a protein hydrolysate commonly used as a brown, liquid condiment in Asian countries [1]. It is rich in valuable nutritional composition and is considered an important dietary source of salt-soluble protein in the form of amino acids [2,3]. However, fish sauce is reported to contain considerable amounts of histamine (>1000 mg/L) [4,5], while the histamine content in fish sauce should be controlled below 50 mg·kg^−1^ in seafood products as suggested by the Food and Drug Administration and below 400 mg·kg^−1^ according to European regulation (EC) No 2073/2005. Excessive histamine intake can cause harmful physiological effects and even life-threatening effects due to histamine’s psychoactive and vasoactive properties [6,7,8,9]. Unfortunately, histamine is difficult to remove once it has formed in food products, even with heat treatments such as autoclaving (121 °C for 15–20 min) [10]. Previous studies have attempted to solve the histamine removal problem by using starter cultures that possess amine oxidase activity, which is responsible for detoxification and can inhibit biogenic amine accumulation during fish sauce fermentation [1,11,12]. 

In the present study, we first proposed a method of reducing histamine content in the fish sauce using electrodialysis (ED). ED is an electrochemical separation technique that uses electric potential as a driving force to move ions through selective ion-exchange membranes and separate them from other molecules in aqueous solutions [13,14,15]. For example, ED can be used for the separating of volatile fatty acids, lactic acid, amino acid, and salt [16,17,18,19]. Existing research about fish sauce using ED applications focused on desalination [20,21,22,23]. However, there is still no report using the ED method to remove histamine from fish sauce.

Histamine has two basic centers, one at the side chain amino group and the other at the acidic imidazole nitrogen moiety, with pKa values of 6.9 and 10.4, respectively [24,25]. Histamine may occur in various forms due to its various states of ionization, tautomeric properties, and various side-chain conformations [26]. A slight change in pH from 6 to 7 might change histamine from a charge acceptor to a charge donor [24]. Therefore, it is theoretically feasible to remove histamine using ED. In this work, the removal of histamine from fish sauce was carried out using ED, which is expected to provide a new strategy for controlling biogenic amines in liquid food products. The effects of operating parameters including input current, pH, and flow velocity on the removal efficiency of histamine in fish sauce were optimized by response surface methodology (RSM). Considering ED may also remove other components in the product [27,28], it is also important to be concerned about the loss of other nutrients such as amino nitrogen (ANN), salts, and volatile compounds during this process. Therefore, the effects of ED on the ANN content, sodium chloride (NaCl) content, and flavor profile of fish sauce were also determined in the present study.

## 2. Materials and Methods

### 2.1. Fish Sauce Properties

Fish sauce was provided by Zhejiang Industrial Group Co., Ltd. (Zhoushan, China). The fermentation processes of fish sauce were as follows: one kilogram of frozen anchovy (*Engraulis ringens*) was thawed at refrigeration temperature, mixed with 8% starter culture *Aspergillus oryzae* and salt (16%), and kept at 30 °C for 5 days to be liquefied by autolysis. Thereafter, the sample was processed by a 3-month fermentation at 30 °C, sterilization, and filtration. The sample was transported to the laboratory in a tightly sealed plastic bottle and stored at room temperature. The main physicochemical properties of the fish sauce were as follows: 81.56 ± 2.52 mg/100 mL histamine content, 1.25 ± 0.13 g/100 mL ANN content, 21.52 ± 0.23% NaCl content, and pH 5.1 ± 0.1.

### 2.2. Electrodialysis Equipment and Procedures

**Figure 1:**
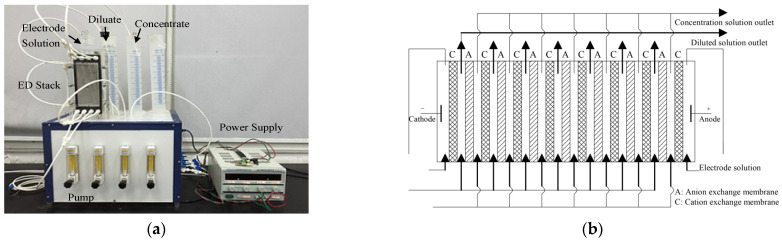
The layout of the ED system (**a**) and a close-up of the ED stack (**b**). The system layout includes a power supply, ED stack, pump, and three containers with electrode solution, concentrate and diluate. The ED stack contains cation- and anion-exchange membranes in alternating series between two electrodes.

A laboratory-scale ED unit (Circle-tech, Zhejiang, China) was used. The ED system layout and an ED stack close-up are illustrated in Figure 1. The equipped membrane stack consists of seven cell pairs, which are formed by eight sulfonic acid-type cation exchange membranes and seven quaternary amine-type anion exchange membranes in an alternating pattern between two electrode compartments. The total effective membrane area is 200 cm^2^. The electrodes consist of a titanium-plated ruthenium dioxide anode and cathode.

ED experiment was carried out in batch mode. An amount of 1% [w∙w^−1^] Na_2_SO_4_, fish sauce, and water were put into the containers labeled as electrode solution, diluate and concentrate in Figure 1a, respectively, and the solutions were then circulated through the corresponding compartments of the stack. A DC power supply (Longwei, TPR–3010D, HK) applied electric potential to both electrodes, and the electric current and voltage were displayed. The limiting current of the ED system used for fish sauce was 8.0 A.

### 2.3. Univariate Analysis

To evaluate the effects of input current (3.0, 4.0, 5.0, 6.0, and 7.0 A), pH (3.0, 4.0, 5.0, 6.0, and 7.0), and flow velocity (10, 20, 30, 40 and 50 L∙h^−1^) on histamine removal, ANN loss and NaCl reduction during ED, a univariate analysis was conducted. For input current analysis, the flow velocity was set to 40 L∙h^−1^ and the ED system voltages were recorded every 2 min. For pH analysis, the constant current and flow velocity were set to 5.0 A and 40 L∙h^−1^, respectively, and the pH of the fish sauce was adjusted with 0.5 M hydrochloric acid and 0.5 M sodium hydroxide. For flow velocity analysis, the input current was set to 5.0 A. ED was operated at ambient temperatures for 50 min. For all the batches, the final fish sauce volumes were recorded, and samples were taken for the analysis of histamine, ANN, and NaCl concentrations.

### 2.4. Response Surface Methodology (RSM) Analysis

A fractional 3-level–3-factor experimental design with three replicates at the center point was adopted [29]. Three factors (input current, pH, and flow velocity), levels and the experimental design in terms of coded and uncoded were given in Table 1. The levels of the three factors were set according to univariate analysis results. Histamine removal and ANN loss rates were taken as the response (Y).

A second-order polynomial equation was fit to the data by a multiple regression procedure, which resulted in an empirical model that related the measured response to the independent variables of the experiment. The model equation is:

Y = a_0_ + a_1_X_1_ + a_2_X_2_ + a_3_X_3_ + a_11_X_1_^2^ + a_22_X_2_^2^ + a_33_X_3_^2^ + a_12_X_1_X_2_ + a_23_X_2_X_3_ + a_13_X_1_X_3_ (1)

where Y is the predicted response; a_0_, the intercept; a_1_, a_2_, a_3_, the linear coefficients; a_11_, a_22,_ a_33_, the squared coefficients; and a_12_, a_23,_ a_13_, the interaction coefficients. Design Expert Software (Version 8.0, StatEase, Inc., Minneapolis, MI, USA) was used to generate response surface graphs. Optimal operation conditions of ED to maximize histamine removal rate and minimize ANN loss rate was obtained, and results were calculated using Equation (1). To verify the model, the experiment was carried out with the optimized operation conditions, and the relevant experimental values were analyzed and compared to theoretical values.

### 2.5. Quantification of Histamine

Histamine content was measured according to the method of Zhou et al. with minor modifications [30]. In brief, 0.4 M perchloric acid was used to extract histamine from 2 mL fish sauce aliquots. A total sample solution volume of 25 mL was obtained. After derivatization with dansyl chloride, the histamine concentration was detected by HPLC (Waters e2695, Milford, MA, USA). A Waters C18 column (5 µm, 4.6 × 250 mm) was used with ammonium acetate (0.01 M, solvent A) and water–acetonitrile (1:9) (contained 0.01 M ammonium acetate, solvent B) as the mobile phases with a flow rate of 1 mL∙min^−1^. The sample injection volume was 10 μL, and the sample was monitored at 254 nm. The histamine reduction rate after ED was calculated via the following formula:

(2) $$\mathrm{Histamine}\;\mathrm{reduction}\;\mathrm{rate}\;(\%)=\frac{\mathrm{histamine}\;\mathrm{concentration}\;\mathrm{before}\;\mathrm{ED-histamine}\;\mathrm{concentration}\;\mathrm{after}\;\mathrm{ED}}{\mathrm{histamine}\;\mathrm{concentration}\;\mathrm{before}\;\mathrm{ED}}\times 100\%$$

### 2.6. Quantifications of NaCl and ANN

The sodium chloride concentration was determined in triplicate by titration with NH_4_CNS after precipitation of AgCl by surplus AgNO_3_ (AOAC 937.09). ANN concentrations were determined according to Zhou et al. [30]. In brief, 20 mL diluted fish sauce samples were mixed with 60 mL distilled water and 20 mL formalin solution (40%), then titrated to pH 9.6 with 0.1 M sodium hydroxide. The volume of consumed sodium hydroxide was recorded to determine ANN concentration. The reduction rates of NaCl and ANN after ED were calculated by the following formula, respectively:

(3) $$\mathrm{NaCl}/\mathrm{ANN}\;\mathrm{reduction}\;\mathrm{rate}\;(\%)=\frac{\mathrm{NaCl}/\mathrm{ANN}\;\mathrm{concentration}\;\mathrm{before}\;\mathrm{ED-NaCl/ANN}\;\mathrm{concentration}\;\mathrm{after}\;\mathrm{ED}}{\mathrm{NaCl}/\mathrm{ANN}\;\mathrm{concentration}\;\mathrm{before}\;\mathrm{ED}}\text{×}100\%$$

### 2.7. Analysis of Amino Acid Composition

Amino acid composition of fish sauce before and after ED was determined according to the method of Lu et al. with a slight modification [31]. An amount of 1 mL of the fish sauce and 9 mL of 1% sulfosalicylic acid were mixed in a centrifuge tube, stood for 15 min, and centrifuged at 3000 rpm for 15 min. The supernatant was collected and passed through a 0.45 μm aqueous membrane. The amino acid composition of the supernatant was quantitatively analyzed by an amino acid automatic analyzer (S-433D, Sykam, Germany). The reduction rate of amino acids after ED was calculated by the following formula:

(4) $$\mathrm{amino}\;\mathrm{acid}\mathrm{content}\;\mathrm{reduction}\;\mathrm{rate}\;(\%)=\frac{\mathrm{amino}\;\mathrm{acid}\;\mathrm{content}\;\mathrm{before}\;\mathrm{ED-amino}\;\mathrm{acid}\;\mathrm{content}\;\mathrm{after}\;\mathrm{ED}}{\mathrm{amino}\;\mathrm{acid}\;\mathrm{content}\;\mathrm{before}\;\mathrm{ED}}\text{×}100\%\;$$

### 2.8. Analysis of Volatile Compounds

Volatile compounds measurements were carried out by Finnigan TRACE GC 2000 GC-MS (Thermo Finnigan, San Jose, CA, USA) equipped with a DB-WAX column (30 m length × 0.25 mm × 0.25 µm) according to the method of Hang et al. [32]. An amount of 5mL of fish sauce samples were placed in a 15mL vial and fitted with a PTFE silicone septum. A preconditioned solid-phase microextraction fiber coated with 75 µm carboxen/polydimethylsiloxane (Supelco, Bellefonte, PA, USA) was inserted into the headspace of the sample bottle and incubated at 55 °C for 30 min. Then the fiber was inserted into the GC injector for desorption for 5 min at 250 °C. The temperature of the GC oven was set at 40 °C for 5 min, then raised up to 220 °C (held for 10 min) by 6 °C/min and a stable speed of 0.8 mL/min. Mass spectrometer conditions were as follows: mass range was 40–450 amu, ionization energy was 70 eV, mass selective detection (MSD) ion source temperature was 250 °C, and detector temperature was 250 °C. The separated data were compared and identified with MST02 library. The relative content was quantitatively analyzed with mean peak area values using the internal standard method, with TMP used as the internal standard.

### 2.9. Statistical Analysis

For univariate analysis, all experiments were performed in triplicate, and the results were presented as the mean ± standard deviation (SD) of replicated measurements. One-way analysis of variance (ANOVA) was performed using the SPSS 21 computer program (SPSS Inc., Chicago, IL, USA), and differences in mean values were determined with the least significant difference (LSD, *p* < 0.05) procedure of the statistical analysis system.

## 3. Results and Discussion

### 3.1. The Effect of Input Current on Histamine Removal from Fish Sauce

**Figure 2:**
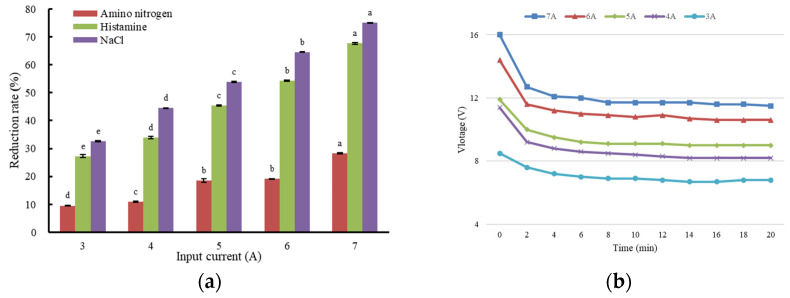
The effect of input current on histamine, ANN, and NaCl reduction rates (**a**) and ED voltage (**b**). In panel (**a**), data were expressed as the reduction rates of their contents before ED. Different letters above the bars indicate significant differences among treatments (*p* < 0.05).

Histamine, ANN, and NaCl reduction rates of fish sauce after ED under different input currents are shown in Figure 2a Increases in input current significantly increased the histamine and NaCl reduction rates of fish sauce (*p* < 0.05) (Figure 2a); however, the ANN loss also increased simultaneously. Increasing the input current from 3.0 to 7.0 A increased the histamine removal rate from 27.25% to 67.63%, ANN loss rate from 9.54% to 28.31%, and NaCl reduction rate from 33% to 75% (Figure 2a). The same increase in input current also increased the voltage of the ED system (*p* < 0.05) (Figure 2b).

This result was consistent with the reports of Xu et al. [33], who found that the increase in voltage also increased the ED current density. ED efficiency was associated with the ion transport properties through the ion-exchange membranes in a previous report [34]. The transport of ions was proportional to the quantity of electricity flowing through the circuit [35]. The applied current has been indicated as a dominant factor in ED performance and higher currents without exceeding the limiting current density and could accelerate the separation efficiency [36,37]. The excessive current will lead to a decrease in ED efficiency [38]. These observations support our results that the histamine removal rate increased with the electric current of the ED. The increased reduction rate of NaCl content was also consistent with the reports of Banasiak et al. [39] and Chindapan et al. [20], who found that higher voltages resulted in higher desalination with groundwater and fish sauce samples during the ED process.

### 3.2. The Effect of Initial pH Value on Histamine Removal from Fish Sauce

**Figure 3:**
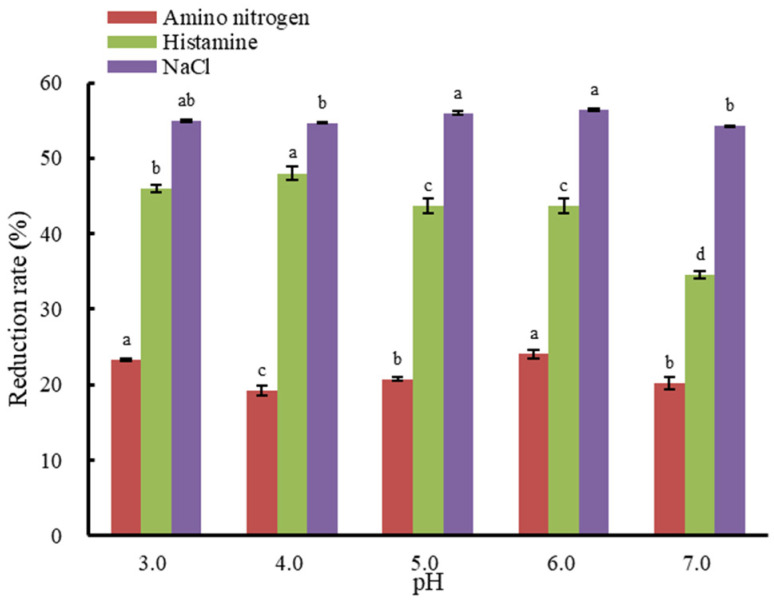
The effect of pH on histamine, ANN, and NaCl reduction rates. Data were expressed as the reduction rates of their contents before ED. Different letters above the bars indicate significant differences among treatments (*p* < 0.05).

Figure 3 shows the effects of the initial fish sauce pH value on histamine, ANN, and NaCl reduction rates. The results showed that the histamine removal rate reached the maximum value of 48.41% at pH 4.0 and the minimum value of 35.50% at pH 7.0, the ANN loss rate was relatively lower when the pH value was 4.0 or 5.0, while the NaCl reduction rate was about 55% at any pH value.

The effect of the initial pH value on histamine removal suggested that the behavior of the ionic molecules in fish sauce was pH dependent. It has been reported that histamine becomes protonated at specific sites [25]. In strongly basic environments above pH 10.4, histamine will be present predominantly in its neutral form. Upon lowering the pH, the aliphatic amino group will bind to a proton, creating a single-protonated histamine or histamine+. At even lower pH, the imidazole ring can bind to a proton and form a double-protonated form of histamine, histamine++ [9]. This double-protonated histamine is the dominant form in solutions with pH values below 6.9 [25]. More highly charged particles at constant current may result in higher migration rates in ED; hence, we found in the present study that the histamine removal efficiency in fish sauce was the highest at pH 4.0. Meanwhile, at pH 7.0, histamine was present mostly in its single protonated form. This could explain why the histamine removal rate of fish sauce showed a significant decrease at pH 7.0 than that at pH 4.0 (*p* < 0.05).

The reason behind amino acid electromigration is the existence of cations and anions at equilibrium with bipolar ions as a result of their interactions with H^+^ and OH¯ ions of dissociating water [16,40]. In amino acid electromigration, pH changes play an important role in terms of the efficiency of the process [41,42]. Water splitting could result in a pH change to affect the charge behavior of the amino acids [43]. Initial pH values could affect the migration rates of amino acids by altering their charged properties. It was reported that keeping the feed pH at the isoelectric point of amino acids helped most of them to exist as bipolar ions and stay in the feed without migrating into the adjacent compartment [16]. Thus, it was speculated that the isoelectric point of the complex amino acid composition in fish sauce was approximately 4.0 or 5.0, as it was at this point that the ANN loss rate was relatively lower (Figure 3). Researchers have been conducting research into reducing the loss of amino acids in the feed after ED treatment. It was reported that the “barrier effect” would limit amino acid transport through both cation- and anion-exchange membranes because of the interactions between amino acid ions and dissociating water [40,44]. Wang et al. introduced a new ED technique with porphyrin thin-film composite cation exchange membranes to prevent amino acid loss while achieving efficient desalination [28]. In addition, efforts have also been attempted towards reducing amino acid loss using pH regulation. Shen et al. proposed that during glutamine fermentation broth desalination with an ED process, the loss of glutamine could be reduced effectively if the pH was controlled near the isoelectric point of glutamine [45].

### 3.3. The Effect of Flow Velocity on Histamine Removal from Fish Sauce

**Figure 4:**
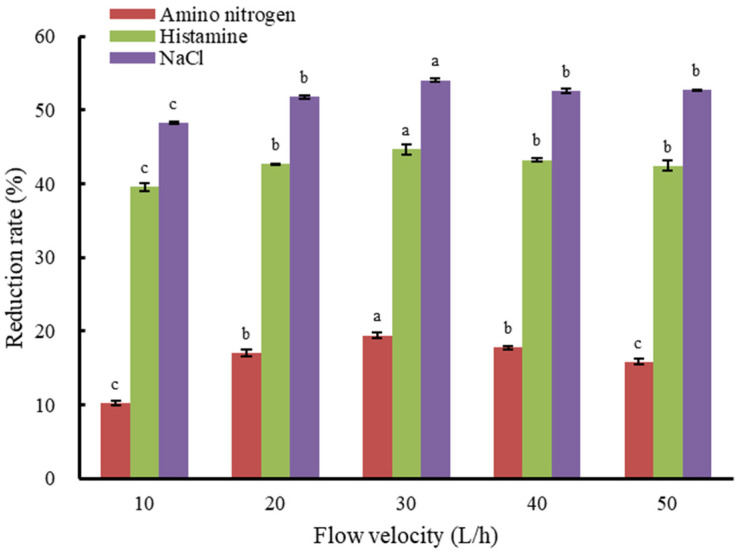
The effect of pH on histamine, ANN, and NaCl reduction rates. Data were expressed as the reduction rates of their contents before ED. Different letters above the bars indicate significant differences among treatments (*p* < 0.05).

Changes in histamine removal, ANN loss, and NaCl reduction rates of fish sauce after ED at various flow velocities were shown in Figure 4. The results showed that all of them increased first and then decreased with the increasing flow velocity. The higher amino acid fluxes could decrease the current efficiency, resulting in a lower amino acid loss rate [43]. The histamine removal rate reached a maximum value of 44.67% at the flow velocity of 30 L∙h^−1,^ and the ANN loss rate reached a maximum value of 19.42% at the flow velocity of 30 L∙h^−1^. In addition, the NaCl reduction was stable and kept at a rate above 53% at a flow velocity higher than 20 L∙h^−1^.

The increases in removal rates of histamine, ANN, and NaCl could be explained by two effects. Generally, a higher flow rate produces a higher degree of turbulence and thinner hydrodynamic boundary layers. Since a significant part of the stack resistance can be traced to the ion-depleted boundary layers [46], operations at high fluid velocities may result in a substantial decrease in stack resistance and a more efficient histamine removal. In addition, high flow rates can inhibit membrane fouling and have a scrubbing effect on the membrane surface, sweeping away proteins and colloidal particles that might otherwise foul the membranes [46]. Different flow velocities at the membrane surface would also cause different residence times [47]. When the flow velocity values continued to increase, the residence time decreased. Therefore, the exchange of ions might not be sufficient, resulting in a significant decrease in histamine removal rate, ANN loss rate, and NaCl reduction rate (*p* < 0.05). This was in accordance with the report of Sadrzadeh and Mohammadi [48], where the separation rate values fell, and ED performance decreased at higher flow rates. However, in some reports, flow velocity had little effect on separation ED efficiency [49,50].

### 3.4. Optimization of ED Operation Conditions by RSM and Model Validation

To minimize ANN loss while improving the histamine removal rate during ED, input currents between 4.0 and 6.0, pH values ranging from 3.0 to 5.0, and flow velocities ranging from 20–40 L∙h^−1^ were chosen for RSM analysis. The experimental plan and the results of BBD experiments studying the effects of the three independent variables are presented in Table 2. The regression equations obtained after the ANOVA gave the histamine removal rate and ANN loss rate as a function of input current, initial pH value, and flow velocity values.

The rate of histamine removal may be best predicted by the model: Y_1_ = 47.49 + 8.06X_1_ − 6.59X_2_ + 2.37X_3_ − 3.73X_1_X_2_ + 0.68X_1_X_3_ − 1.90X_2_X_3_ − 4.89X_1_^2^ − 4.54X_2_^2^ + 0.12X_3_^2^, where Y_1_ is the histamine removal rate (%); X_1_ is the input current (A); X_2_ is the pH; and X_3_ is the flow velocity (L∙h^−1^).

The rate of ANN loss may be best predicted by the model: Y_2_ = 16.04 + 2.78X_1_ − 0.82X_2_ − 1.58X_3_ − 0.10X_1_X_2_ − 0.38X_1_X_3_ + 0.09X_2_X_3_ − 0.30X_1_^2^ + 2.52X_2_^2^ + 0.71X_3_^2^, where Y_2_ is the ANN loss rate (%); X_1_ is the input current (A); X_2_ is the pH; and X_3_ is the flow velocity (L∙h^−1^).

Other model results and the optimum conditions were determined using Design Expert function and presented in Table 3 and Table 4, respectively. The coefficient of determination (R^2^) was calculated to be 0.9872 for histamine removal rate and 0.9853 for ANN loss rate (Table 3), indicating that the equation was highly reliable. When expressed as a percentage, R^2^ is interpreted as the rate variability in the response explained by the statistical model. This implied that the sample variations of 98.72% for histamine removal rate and 98.53% for ANN loss rate were attributed to the independent variables, indicating a satisfactory adjustment of the quadratic model to the experimental data.

Adequate precision measures the signal-to-noise ratio, and ratios greater than 4 are desirable. A precision of 20,918 and 19,984 for histamine removal rate and ANN loss rate, respectively, was calculated, indicating an adequate signal. Adjusted R^2^ could correct the R^2^ value for the sample size and the number of terms in the model. In this case, the adjusted R^2^ values, 0.9643 and 0.9588 for histamine removal and ANN loss rates, respectively, were close to their corresponding R^2^ values, demonstrating that the sample size was large enough for the terms in the model. For histamine removal rate, all of the linear coefficients (X_1_, X_2_, X_3_), one cross-product term (X_1_X_2_), and two quadratic terms (X_1_^2^ and X_2_^2^) are significant model terms (*p* < 0.05), and for ANN loss rate, all of the linear coefficients (X_1_, X_2_, X_3_) and one quadratic term (X_2_^2^) are significant model terms (*p* < 0.05), demonstrating that the response values to experimental factors are not simple linear relationships.

The computed F-value, 43.01 for histamine removal and 37.22 for ANN loss, implied that the model is highly significant (*p* < 0.05). The model also showed a statistically insignificant lack of fit, as is evident from the “Lack of Fit F-value” of 4.08 and 0.64 for histamine removal and ANN loss with the relative ‘Prob > F’ > 0.05.

**Figure 5:**
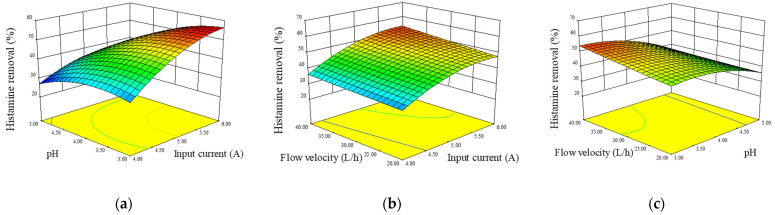
Response surface plots showing the interaction effects of input current and pH at a constant flow velocity of 30 L/h (**a**), input current and flow velocity at a constant pH value of 4.0 (**b**), and pH value and flow velocity at a constant input current of 5 A (**c**) on histamine removal. The change in color from blue to red indicates the reduction of histamine from less to more, and higher slopes result from greater changes.

**Figure 6:**
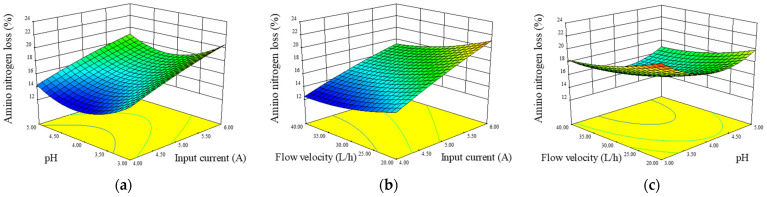
Response surface plots showing the interaction effect of input current and pH at a constant flow velocity of 30 L/h (**a**), input current and flow velocity at a constant pH value of 4 (**b**), and pH value and flow velocity at a constant input current of 5 A (**c**) on ANN loss. The change in color from blue to red indicates the loss of amino nitrogen from less to more, and higher slopes result from greater changes.ANOVA showed that X_1_X_2_ was significant (*p* < 0.05) for histamine removal rate, whereas for ANN loss rate, no significant cross-product was found (*p* > 0.05). As seen in Figure 5a, the histamine removal rate increased slightly with an increase in pH at a low constant current. However, when the current increased, the surface increased more densely with the decrease in pH value, which means that the function of pH value on histamine removal rate increased. This phenomenon might be because higher input currents lead to higher histamine molecule transport rates, while lower pH values resulted in more charged histamine ions.

The 3-D response surface curve was plotted using the statistically significant model to understand the interactive effects of input current, pH, and flow velocity on histamine removal and ANN loss. The interactive effects of any two variables on histamine removal rate are depicted in Figure 5a–c. The input current showed the most significant effect on histamine removal, as the curve increased steeply with the increase in input current. This was in accordance with the results in univariate analysis. The interactive effect of variables on ANN loss rate is shown in Figure 6a–c, and the input current of ED system was again found to have the most significant effect.

The optimum operating conditions of the ED system should provide a maximum histamine removal rate and a minimum ANN loss rate. The optimum conditions were determined using the Design Expert function and presented in Table 4. The point at an input current = 5.1 A, pH value = 3.8, and flow velocity = 40 L∙h^−1^ could be recommended as the practical optimum. Under this condition, the predicted histamine and ANN reduction rates were 52.62% and 15.72%, respectively. This set of conditions could also be used to test the suitability of the model equation for predicting the response values. The experimental results, with a histamine removal rate of 53.41% and an ANN reduction rate of 15.46%, were found to be in good agreement with the predicted values. To evaluate the safety and quality, the histamine residual amount, 38.00 mg/100 mL, meet the criterion of Commission Regulation (EC) No 2073/2005, and the ANN residual amount, 1.06 g/100 mL, was still higher than the best level of the Chinese industrial standard SB/T 10324-1999 for fish sauce. Applying RSM to optimize the ED operation process has been carried out in many studies [51,52,53]. RSM was effective in providing comprehensive and informative insight into the system, by reducing the amount of time and effort required for the investigation of multi-factor, multi-response systems, leading to faster process optimization [54,55]. Moreover, it was worth noting that the NaCl content of the fish sauce was found to have decreased by 56.23% after ED processing under optimal operation conditions. This means that in addition to removing histamine, the ED technique also desalinated the fish sauce efficiently at the same time.

### 3.5. ANN Loss Rate

ANN content was an important indicator for evaluating the quality of fish sauce. ANN was generally nitrogen in the form of free amino acids and the level of ANN could basically reflect the level of free amino acids. Amino acids were ampholytes whose charge was influenced by pH value. When the feed pH was greater than its isoelectric point, amino acids existed in the form of anion and moved to the anode through the anion membrane in the direct current electric field. Conversely, amino acids existed in the form of cation and moved to the cathode through the cation membrane. In the process of purifying a single amino acid solution, the feed pH was usually adjusted to the isoelectric point of the amino acid for ED. For example, Wang et al. controlled the pH of soy sauce in ED to 5.71, the average isoelectric point of amino acids, and therefore the amino acid loss rate was the lowest [42]. However, the migration of amino acids in ED cannot be avoided. Changes in amino acids of fish sauce before and after ED at the optimal level and ANN loss rate were given in Table 5. Table 5 showed that the total amino acid loss rate was 15.46% under the optimized conditions. There was a large amount of loss rate of histidine, arginine, and tyrosine, which might be related to their corresponding side chains, imidazole, amino, and guanidine groups, respectively. The imidazolium group has a pKa value close to 7 [56], and the pKa values of the conjugate acids of amino and guanidine groups are much higher than 7 [57,58]. Thus, under the optimal conditions when pH was 3.8, the side chains of histidine, arginine, and tyrosine were easy to be protonated. Further research is needed to minimize the loss of basic amino acids during ED, such as choosing a suitable ion exchange membrane that is selective for ions and even adsorbs them [16].

### 3.6. Variations in Volatile Compounds

Volatile compounds of fish sauce were significantly changed after ED under the optimal operation conditions, which is shown in Table 6. After ED, alcohols and aldehydes decreased, while ketones, acids, and pyrazines increased. Aldehydes dominated and were identified as the main components of the fishy smell [59]. As the relative content of aldehydes decreased, the most obvious change in fish sauce was to be less fishy after ED. In addition, the floral and oily aroma of fish sauce was weakened after the ED as alcohols decreased apparently [60,61]. Ketones and carboxylic acids mainly contributed to cheesy notes [61,62], and their relative rate increased sharply after ED. However, due to their high odor threshold value, the changes in ketones and carboxylic acids had few impacts on the flavor of fish sauce [63,64]. The increased, 2, 6-dimethylpyrazine and 2-ethyl-6-methylpyrazine, would contribute to the cooked rice and buttery popcorn aroma [60] produced from Maillard reactions during fermentation [65]. Other compounds decreased obviously, and most of them were heterocyclic sulfur compounds that had barbecue flavors [66]. In addition, the rapid growth of some volatile compounds such as 2-ethyl-4-glutenal, 2, 3-dimethylglutaraldehyde, and hexanoic acid during ED may be caused by electrocatalysis activities [67], and further studies were needed to explore the electrocatalysis procedures in ED.

## 4. Conclusions

ED was proven to be a feasible technique for removing histamine from fish sauce. Based on histamine removal and ANN loss rates of the univariate experiments, the optimal ranges for electroosmotic input current, pH, and flow velocity were found to be 4.0–6.0 A, 3.0–5.0, and 20–40 L∙h^−1^, respectively. RSM further determined the best operation conditions for input current, pH, and flow velocity were 5.1 A, 3.8, and 40 L∙h^−1^, respectively. Under these conditions, the histamine removal rate reached 53.41% and the histamine level met the safety criteria in Regulation (EC) No 2073/2005, while the ANN loss rate declined to 15.46% and the fish sauce was still on the best quality level according to Chinese standard SB/T 10324–1999. Moreover, the changes in NaCl content and volatile compound composition showed that the fish sauce was less salty and less fishy after ED, which could provide better acceptability.

## Figures and Tables

**Table 1:** ED process variables and their levels for Box–Behnken design (BBD) ^a^.

Variables	Symbols		Levels		
	Coded	Uncoded	−1	0	1
**Input current (A)**	X_1_	x_1_	4	5	6
**pH**	X_2_	x_2_	3.0	4.0	5.0
**Flow velocity (L∙h^−1^)**	X_3_	x_3_	20	30	40

^a^ X_1_ = x_1_ − 5, X_2_ = x_2_ − 4, and X_3_ = (x_3_ − 30)/10.

**Table 2:** BBD treatment schedule and the response in terms of histamine removal and ANN loss.

Experiment No.	Input Current (A) (X_1_)	pH (X_2_)	Flow Velocity (L∙h^−1^) (X_3_)	Histamine Removal (%) (Y_1_)	ANN Loss (%) (Y_2_)
1	0	−1	1	53.67	18.27
2	1	1	0	34.63	20.27
3	0	0	0	48.57	16.60
4	0	1	−1	36.25	20.09
5	0	0	0	46.56	15.31
6	−1	−1	0	34.01	16.05
7	−1	0	1	35.42	12.78
8	0	0	0	47.32	16.19
9	1	−1	0	55.16	22.38
10	−1	0	−1	31.45	15.14
11	−1	1	0	28.41	14.33
12	1	0	1	55.34	17.00
13	1	0	−1	48.65	20.87
14	0	−1	−1	45.74	21.65
15	0	1	1	36.59	17.07

**Table 3:** ANOVA for the response surface quadratic model.

Values	Histamine Reductio Rate	ANN Reduction Rate
R^2^	0.9872	0.9853
Adj R^2^	0.9643	0.9588
Adeq precision	20.918	19.918
Model F-value	43.01 ^a^	37.22 ^a^
Lack of fit F-value	4.08 ^b^	0.64 ^b^
Pure error	1.03	0.43

^a^ Significant at 0.1%; ^b^ insignificant.

**Table 4:** Feasible optimum conditions and the predicted versus experimental response values.

Optimum Condition	Coded Levels	Actual Levels
Input current	0.1	5.1 A
pH	−0.2	3.7
Flow velocity	1	40 L∙h^−1^
Responses	Predicted values	Experiment values
Histamine reduction rate	52.62%	53.41% ± 0.41%
Histamine content after ED (mg/100 mL)	38.74	38.00 ± 0.33
ANN reduction rate	15.72%	15.46% ± 0.28%
ANN content after ED (g/100 mL)	1.035	1.06 ± 0.00
Salt reduction rate after ED	-	56.23 ± 0.1%

Data are expressed as the means ± standard deviation (n = 3).

**Table 5:** Changes in amino acids in fish sauce before and after ED.

	Isoelectric Point	Before ED Content (g∙L^−1^)	After ED	Reduction Rate (%)
			Content (g∙L^−1^)	
Aspartic acid	2.77	7.96 ± 0.11 d	7.62 ± 0.19 d	4.27
Threonine	6.16	3.79 ± 0.03 h	3.48 ± 0.09 g	8.18
Serine	5.68	3.9 ± 0.05 h	3.6 ± 0.07 g	7.69
Glutamic acid	3.22	9.01 ± 0.07 c	8.17 ± 0.11 c	9.32
Glycine	5.97	2.64 ± 0.02 k	2.37 ± 0.04 i	10.23
Alanine	6.00	6.01 ± 0.04 e	5.52 ± 0.07 e	8.15
Cysteine	5.05	1.78 ± 0.01 m	1.67 ± 0.03 j	6.18
Proline	5.96	4.82 ± 0.03 c	4.47 ± 0.06 b	7.26
Methionine	5.74	2.59 ± 0.02 k	2.39 ± 0.03 i	7.72
Isoleucine	6.02	3.17 ± 0.04 j	2.87 ± 0.04 h	9.46
Leucine	5.98	4.99 ± 0.05 f	4.51 ± 0.11 f	9.62
Tyrosine	5.68	0.32 ± 0.01 o	0.26 ± 0.05 k	18.75
Phenylalanine	5.48	2.27 ± 0.03 l	2.04 ± 0.07 i	10.13
Histidine	7.59	3.4 ± 0.06 i	2.35 ± 0.16 i	30.88
Lysine	9.74	31.02 ± 0.21 a	24.7 ± 0.49 a	20.37
Arginine	10.76	13.63 ± 0.16 b	9.55 ± 0.34 bc	29.93
Proline	6.30	1.58 ± 0.01 g	1.4 ± 0.09 j	11.39
Total amino acids		102.88 ± 0.41	86.97 ± 1.07	15.46

Different lowercase letters in the same column represent significant differences at *p* < 0.05.

**Table 6:** Volatile compounds in fish sauce before and after ED.

Compound Name	Relative Content (%)		Significant Differences before and after ED	Relative Change	Odor Description
	Before ED	After ED			
Alcohol	5.54	2.75			
Cyclopentanol	0.01 ± 0.01 d	0.01 ± 0.01 f	A, A (*p* > 0.05)	↘	
2-ethylhexanol	1.96 ± 0.6 d	1.78 ± 0.16 cdef	A, A (*p* > 0.05)	↘	Floral, perfume
phenethyl alcohol	3.35 ± 0.63 cd	0.96 ± 0.16 ef	A, B (*p* < 0.05)	↘↘	Floral, perfume
2-furfuryl alcohol	0.20 ± 0.10 d	0.00 ± 0.00 f	A, A (*p* > 0.05)	↘↘	Oily, burnt sugar
Aldehyde	68.27	59.58			
octanal	0.66 ± 0.37 d	0.66 ± 0.13 ef	A, A (*p* > 0.05)	-	mint, floral, fruit, resin
3-methyl n-butyl aldehyde	1.39 ± 0.42 d	0.69 ± 0.27 ef	A, A (*p* > 0.05)	↘↘	
2-methylpropyl aldehyde	5.22 ± 1.24 cd	3.06 ± 0.39 cdef	A, A (*p* > 0.05)	↘	
3-methyl n-butyl aldehyde	21.37 ± 2.27 a	14.03 ± 3.82 a	A, A (*p* > 0.05)	↘	Almond, nutty, buttery
2-methyl n-butyl aldehyde	10.71 ± 5.81 b	9.70 ± 2.02 b	A, A (*p* > 0.05)	↘	Nutty, buttery, oily
2, 3-dimethylglutaraldehyde	0.24 ± 0.10 d	1.48 ± 0.12 def	A, A (*p* > 0.05)	↗↗	
2-ethyl-4-glutenal	0.00 ± 0.00 d	9.44 ± 1.32 b	A, B (*p* < 0.05)	↗↗	
methylthiopropanal	18.21 ± 5.41 a	13.91 ± 1.93 a	A, A (*p* > 0.05)	↘	Boiled potato
benzaldehyde	2.82 ± 0.08 cd	1.75 ± 0.74 cdef	A, B (*p* < 0.05)	↘	Almond, burnt sugar, sweet
phenylacetaldehyde	4.00 ± 1.19 cd	2.37 ± 0.46 cdef	A, A (*p* > 0.05)	↘	Beer not fresh
phenylglyoxal	1.33 ± 0.19 d	1.20 ± 0.07 ef	A, A (*p* > 0.05)	↘	
pelargonic aldehyde	2.31 ± 0.22 cd	1.29 ± 0.42 ef	A, A (*p* > 0.05)	↘	Green, grassy, moss
Ketone	2.41	6.19			
butanone	2.17 ± 1.02 cd	5.38 ± 1.40 c	A, A (*p* > 0.05)	↗↗	cheesy
5-ethyl-2 (5H) -furanone	0.24 ± 0.02 d	0.81 ± 0.25 ef	A, B (*p* < 0.05)	↗↗	cheesy
Carboxylic acid	9.90	19.32			
N-methyl taurine	0.08 ± 0.03 d	0.57 ± 0.36 ef	A, A (*p* > 0.05)	↗↗	
4-methylvaleric acid	0.47 ± 0.22 d	0.20 ± 0.11 f	A, A (*p* > 0.05)	↘	Pungent sour
hexanoic acid	1.65 ± 0.28 d	9.44 ± 3.55 b	A, A (*p* > 0.05)	↗↗	
3-methylbutyric acid	4.15 ± 0.47 cd	5.35 ± 0.32 cd	A, A (*p* > 0.05)	↗	Dirty socks, sweaty, cheesy
2-methylbutyric acid	1.71 ± 0.15 d	2.20 ± 0.06 cdef	A, A (*p* > 0.05)	↗	Cheesy
2-methylhexanoic acid	1.79 ± 0.79 d	1.47 ± 0.09 def	A, B (*p* < 0.05)	↘	
n-nonanoic acid	0.05 ± 0.03 d	0.08 ± 0.01 f	A, A (*p* > 0.05)	↗	
Pyrazine	2.28	5.79			
methylpyrazine	0.45 ± 0.09 d	0.11 ± 0.04 f	A, A (*p* > 0.05)	↘↘	
2, 6-dimethylpyrazine	1.33 ± 0.57 d	4.43 ± 1.87 cde	A, A (*p* > 0.05)	↗↗	Cooked rice, sweet
2-ethyl-6-methylpyrazine	0.50 ± 0.30 d	1.26 ± 0.40 ef	A, A (*p* > 0.05)	↗↗	
Others	11.60	6.37			
Dimethyl disulfide	3.13 ± 0.79 cd	2.25 ± 0.26 cdef	A, A (*p* > 0.05)	↘	
4-methyl-pyrimidine	0.37 ± 0.05 d	0.95 ± 0.29 ef	A, A (*p* > 0.05)	↗↗	
2, 4-di-tert-butylphenol	0.78 ± 0.14 d	0.23 ± 0.15 f	A, A (*p* > 0.05)	↘↘	
2-acetylpyrrole	7.19 ± 2.08 bc	2.73 ± 0.95 cdef	A, A (*p* > 0.05)	↘↘	
acetophenone	0.06 ± 0.04 d	0.10 ± 0.00 f	A, B (*p* < 0.05)	↗	
2-methyl-naphthalene	0.09 ± 0.04 d	0.10 ± 0.00 f	A, B (*p* < 0.05)	↗	

Different lowercase letters represent significant differences at *p* < 0.05 in the same column. Different upper letters represent significant differences in the same row. The symbol ↘ and ↗ represent the trends of compounds’ relative content in fish sauce after ED, respectively.

## Data Availability

Original data can be obtained from the corresponding author.
